# Isolated Fallopian Tube Torsion: Diagnosis and Management of a Gynecologic Emergency

**DOI:** 10.7759/cureus.46260

**Published:** 2023-09-30

**Authors:** John N Pignataro, Lynnett Schindler

**Affiliations:** 1 Clinical Research, Liberty University College of Osteopathic Medicine, Lynchburg, USA; 2 Obstetrics and Gynecology, Liberty University College of Osteopathic Medicine, Lynchburg, USA

**Keywords:** surgical emergencies, isolated fallopian tube torsion, high clinical suspicion, diagnostic laparoscopy, gynecologic emergencies

## Abstract

Isolated fallopian tube torsion (IFTT) is a rare gynecologic emergency that requires a high index of suspicion and immediate surgical intervention. IFTT is rarely diagnosed preoperatively due to the lack of pathognomonic signs and symptoms.

A 15-year-old female with no medical history presented with acute lower abdominal pain, nausea, and a physical exam significant for diffuse abdominal tenderness to palpation. Ultrasound revealed a large cystic adnexal mass with patent vascular flow to the ipsilateral ovary. Vital signs were stable and laboratory evaluation was unremarkable. Due to an uncertain diagnosis and suspicion of incomplete ovarian torsion, a laparoscopy was performed, which revealed an IFTT and ipsilateral hemorrhagic ovarian cyst. Treatment consisted of unilateral salpingectomy with cystectomy.

IFTT is a surgical emergency with nonspecific signs and symptoms. A high degree of clinical suspicion is needed for prompt management. Diagnostic laparoscopy can be a useful tool in the setting of an uncertain diagnosis.

## Introduction

Adnexal masses are a commonly encountered problem for obstetrician-gynecologists. Most are detected incidentally, and most are benign [1]. Unilateral, intermittent, and then acutely worsening pelvic pain may be suggestive of ovarian torsion and a series of reviews found that 86-95% of patients with an ovarian torsion had an ovarian mass [1,2]. Ovarian torsion is a common surgical emergency with an estimated incidence of 2-15% of women who have surgical treatment of adnexal masses [3]. In this report, we present a case with the provisional and preoperative diagnosis of intermittent or incomplete left ovarian torsion; however, the much less common diagnosis of fallopian tube torsion was made during surgery. Isolated fallopian tube torsion (IFTT) is a rare gynecologic finding that can be difficult to diagnose. IFTT is an emergency requiring immediate surgical intervention.

This article has previously been published as a meeting abstract at the Fourth Annual Liberty University College of Osteopathic Medicine (LUCOM) Research Day event.

## Case presentation

A 15-year-old nulligravida female presented to the emergency department with a two-day history of worsening left pelvic pain and nausea. The pain was described as a persistent dull ache with intermittent stabbing, radiation to the left flank and left shoulder, and made worse by movement. The patient denied passage of stool or flatus over the previous two days. Menstrual cycles were regular and the last menstrual period was two weeks prior. The patient was not sexually active, never had sexual intercourse, and did not have a history of sexually transmitted infection. The physical exam revealed normal vitals with a pulse rate of 64 beats per minute, blood pressure of 113/78 mmHg, respiratory rate of 15 breaths per minute, and an oral temperature of 36.8°C. Of note, all four quadrants were tender to palpation. A pelvic exam performed in the operating room under anesthesia was remarkable for a fixed adnexal mass in the left lower quadrant. The urine pregnancy test was negative.

Labs revealed a normal complete blood count, including a normal white blood cell count, hemoglobin, hematocrit, and platelet count. Additionally, a comprehensive metabolic panel, troponin-I, and D-dimer were within normal limits. Urinalysis was unremarkable and negative for infection. Renal ultrasound, chest X-ray, and kidney, ureter, and bladder (KUB) X-ray were normal. Screening for pelvic infections was not performed on this patient due to her lack of prior sexual activity.

**Figure 1:**
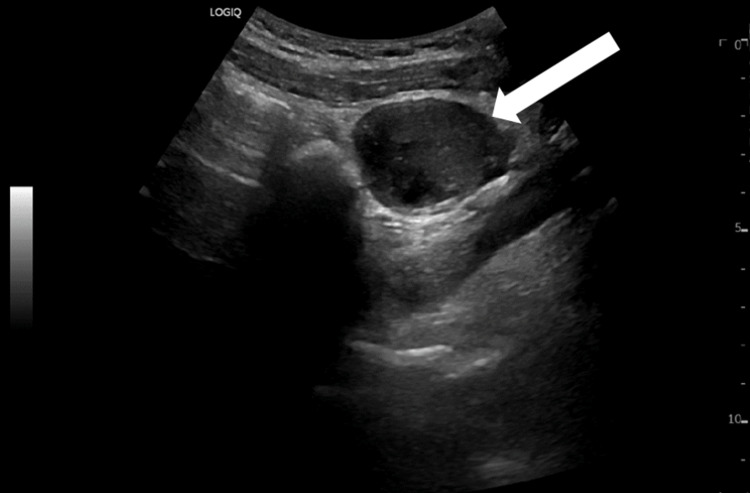
Transverse view of the left ovary.

**Figure 2:**
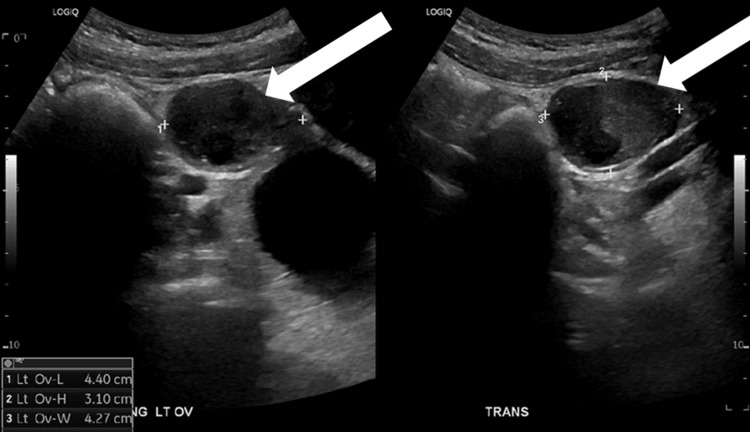
Longitudinal and transverse views of the left ovary with measurements.

**Figure 3:**
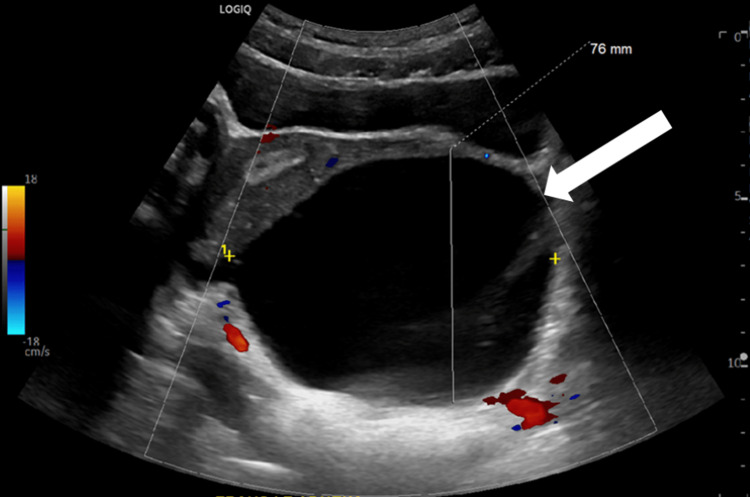
Transverse view of the left adnexa.

**Figure 4:**
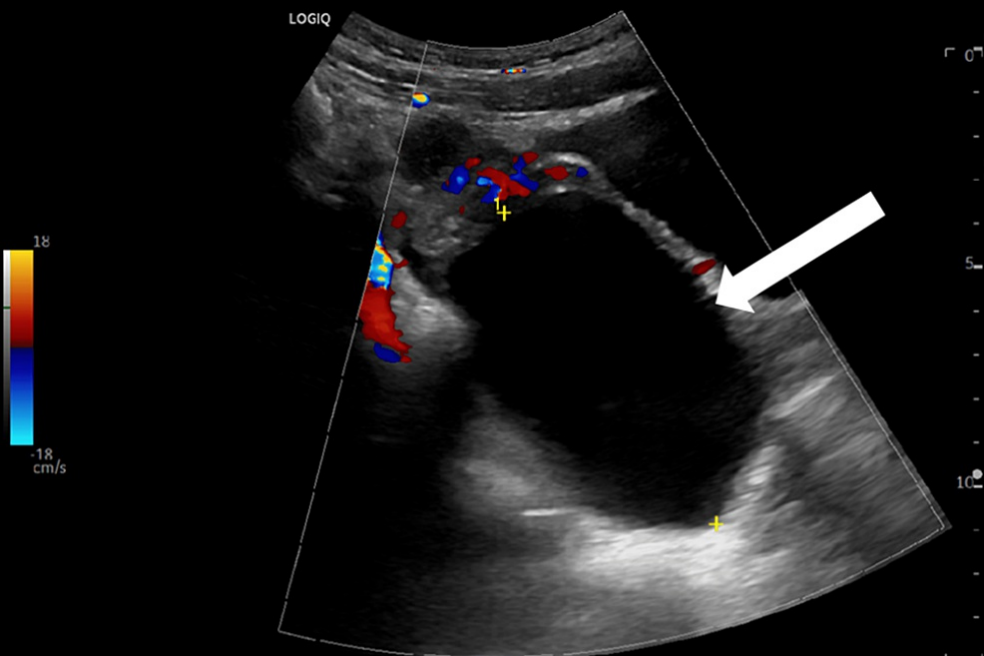
Longitudinal view of the left adnexa.

**Figure 5:**
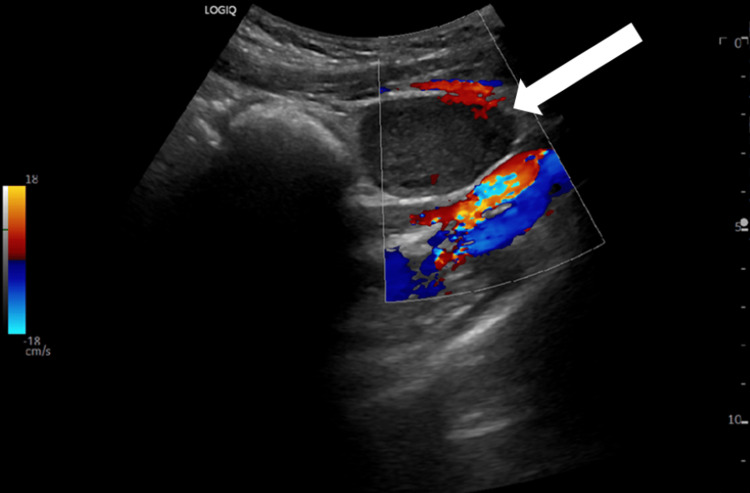
Transverse view of the left ovary with Doppler imaging.

**Figure 6:**
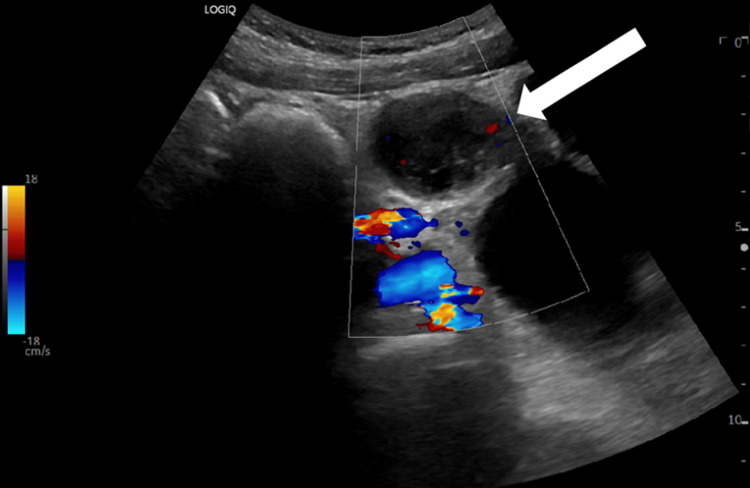
Longitudinal view of the left ovary with Doppler imaging.

Below, Figures 1, 2 are ultrasound images of the pelvis showing a left ovary measuring 4.4 cm x 4.3 cm x 3.2 cm. The ultrasound images in Figures 3, 4 demonstrate a large 10 cm x 8.6 cm x 7.6 cm cystic mass in the left adnexa associated with the left ovary. The ultrasound images in Figures 5, 6 show patent vascular flow to the left ovary on Doppler imaging. The right ovary was not visualized and no free fluid was seen.

The patient’s presentation raised suspicion about ovarian torsion. Diagnostic laparoscopy revealed a left fallopian tube torsion and a left hemorrhagic ovarian cyst. The left fallopian tube was markedly enlarged and hemorrhagic. The right ovary and right fallopian tube were unremarkable. The left fallopian tube was cauterized and transected at the twisting point near the cornual end. The left ovarian cyst, measuring approximately 2 cm x 2 cm x 1 cm, was opened, irrigated, and drained, and the capsule was removed. Pathology report of the left fallopian tube described a hemorrhagic infarction of the fallopian tube. Notably, there were no signs of pelvic adhesions, pelvic masses, or endometriosis. The small intestine, large intestine, and liver appeared unremarkable. The patient tolerated the procedure well without complications. She was discharged on postoperative day one and had fully resumed her activities by the time of her outpatient follow-up two weeks after surgery.

## Discussion

IFTT is a rare occurrence with an estimated incidence ranging from one in 500,000 to one in 1,500,000 women [4,5]. Risk factors for tubal torsion include tubal pathologies such as hydrosalpinx, embryologic abnormalities, broad ligament cyst, para tubal cyst, ectopic pregnancy, pelvic inflammatory disease, abnormal tubal function, adhesions, and endometriosis [2,6]. IFTT can occur without obvious signs or symptoms and is a difficult preoperative diagnosis [4,6]. Other reports describe presentations with similar features to our patient such as lower abdominal/pelvic pain that radiates to the flank, nausea, abdominal tenderness, and palpable adnexal mass [4,6]. Other symptoms include vomiting, dysuria, fever, diffuse or localized abdominal pain, and back pain [6]. Lab evaluation may reveal leukocytosis and ultrasound findings may include ovarian cyst, hydrosalpinx, and free peritoneal fluid [6]. Some investigators report twisting of the vascular bundle can result in a sonographic whirlpool sign specific to tubal torsion [7]. Although a sonographic whirlpool sign was not seen in our patient, normal vascular flow to the ipsilateral ovary, as seen in our patient, is consistent with other reports of isolated tubal torsion [2]. Suspicion for IFTT should also be raised if there is an enlarged tubular structure adjacent to a normal-appearing ovary [8]. In our patient, there was a clearly defined ovary measuring 4.4 x 4.3 x 3.2 cm with an adjacent large 10 x 8.6 x 7.6 cm cystic mass. The presence of a mass distinct from the ipsilateral ovary supports a diagnosis of tubal torsion isolated from ovarian pathology. Ultrasonography is recommended as the first imaging modality; however, accurate diagnosis has been reported to be as low as 30% of patients diagnosed with isolated tubal torsion [6,9]. Our case demonstrates the presence and absence of the various findings suggestive of tubal torsion. These varying clinical presentations and nonspecific findings make the preoperative diagnosis of IFTT challenging.

Fallopian tube torsion is managed surgically. Like ovarian torsion, tubal torsion is managed by untwisting the torsed fallopian tube during laparoscopy [2]. Fallopian tube torsion is a gynecologic emergency and requires immediate intervention [8]. Timely intervention is often delayed and can lead to irreversible damage requiring salpingectomy [4]. Our patient presented two days after the onset of pain and had an extensive and unremarkable evaluation before a pelvic ultrasound revealed the adnexal mass. The fallopian tube was unable to be untwisted during laparoscopy requiring unilateral salpingectomy. There is usually an accompanying cystectomy if there is a benign mass present [2]. Due to the degree the fallopian tube was enlarged, the presence of a para tubal cyst was not diagnosed until the specimen was examined ex vivo. Conservative surgical management with detorsion and cystectomy has been successful for some patients depending on the degree of necrosis [10]. Several studies report low salvageability of the tubes ranging from 12% to 33% [6,11,12]. The length of time since pain onset is an important factor [13]. One case series reports an increased risk of salpingectomy associated with pain lasting more than 24 hours [13]. Our patient presented after 48 hours of worsening pain and histopathology of the fallopian tube demonstrated hemorrhagic infarction and nonviable tissue. Although the left fallopian tube was removed, subsequent fertility should be unchanged with an intact contralateral tube [14]. However, salpingectomy may have an adverse effect on fertility if the remaining tube is damaged by infection, ectopic pregnancy, or torsion, and the patient should be counseled appropriately [6].

IFTT is a rare gynecologic surgical emergency with a difficult preoperative diagnosis that requires prompt intervention. This case demonstrates the nonspecific presentation of IFTT and the need for a high index of suspicion. Soft signs of IFTT seen in this case include acute unilateral abdominal pain, nausea, ileus, diffuse tenderness to palpation, a large cystic adnexal mass, and normal Doppler flow to the ipsilateral ovary [4-6]. Exploratory laparoscopy provided the definitive diagnosis when the preoperative diagnosis was uncertain.

## Conclusions

Fallopian tube torsion has nonspecific signs and symptoms that overlap with other gynecologic pathologies. A high degree of suspicion for ovarian pathology escalated management to diagnostic laparoscopy. Our patient lacked known risk factors for tubal pathology, so clinical judgment was essential to uncover the rare diagnosis of IFTT.
